# Retraction: LncRNA BLACAT1 accelerates non-small cell lung cancer through up-regulating the activation of sonic hedgehog pathway

**DOI:** 10.3389/fonc.2024.1468573

**Published:** 2024-07-30

**Authors:** Frontiers Editorial Office

**Affiliations:** Frontiers Media SA, Lausanne, Switzerland

The journal retracts the April 15^th^ 2021 article cited above.

Following publication, concerns were raised regarding the integrity of the images in the published figures. The authors failed to provide the raw data or a satisfactory explanation during the investigation, which was conducted in accordance with Frontiers’ policies. Given the concerns about the validity of the data, and the lack of raw data, Frontiers no longer has confidence in the findings presented in the article.

This retraction was approved by the Chief Executive Editor of Frontiers. The authors received a communication regarding the retraction and had a chance to respond. This communication has been recorded by the publisher.

